# Development of new copper-64 labeled rhodamine: a potential PET myocardial perfusion imaging agent

**DOI:** 10.1186/s41181-022-00171-2

**Published:** 2022-07-23

**Authors:** Norah AlHokbany, Ibrahim AlJammaz, Basem AlOtaibi, Yousif AlMalki, Bander AlJammaz, Subhani M. Okarvi

**Affiliations:** 1 Chemistry Department, Science College, King Saud University, P.O. Box 22452, Riyadh, 11495 Kingdom of Saudi Arabia; 2 College of Medicine, King Saud University, P.O. Box 22452, Riyadh, 11495 Kingdom of Saudi Arabia; 3 Cyclotron and Radiopharmaceuticals Department, King Faisal Specialist Hospital and Research Centre, P.O. Box 3354, Riyadh, 11211 Kingdom of Saudi Arabia

**Keywords:** Copper-64, Positron emission tomography, Rhodamine, Myocardial perfusion imaging, Radiopharmaceuticals

## Abstract

**Background:**

Myocardial perfusion imaging (MPI) is one of the most commonly performed investigations in nuclear medicine procedures. Due to the longer half-life of the emerging positron emitter copper-64 and its availability from low energy cyclotron, together with its well-known coordination chemistry, we have synthesized ^64^Cu-labeled NOTA- and ^64^Cu-NOTAM-rhodamine conjugates as potential cardiac imaging agents using PET.

**Results:**

^64^Cu-NOTA- and ^64^Cu-NOTAM-rhodamine conjugates were synthesized using a traightforward and one-step simple reaction. Radiochemical yields were greater than 97% (decay corrected), with a total synthesis time of less than 25 min. Radiochemical purities were always greater than 98% as assessed by TLC and HPLC. These synthetic approaches hold considerable promise as a simple method for ^64^Cu-rhodamine conjugates synthesis, with high radiochemical yield and purity. Biodistribution studies in normal Fischer rats at 60 min post-injection, demonstrated significant heart uptake and a good biodistribution profile for both the radioconjugates. However, the ^64^Cu-NOTAM-rhodamine conjugate has shown more heart uptake (~ 10% ID/g) over the ^64^Cu-NOTA-rhodamine conjugate (5.6% ID/g).

**Conclusions:**

These results demonstrate that these radioconjugates may be useful probes for the PET evaluation of MPI.

## Background

Myocardial Perfusion Imaging (MPI) is a non-invasive procedure to provide a sensitive means for detection, localization, and risk satisfaction of ischemic heart disease, assessment of left ventricular function, and myocardial viability. MPI is one of the most commonly performed investigations in nuclear medicine studies. The most widely used MPI is single-photon emission computed tomography (SPECT), usually performed using single-photon radiopharmaceuticals, such as ^99m^Tc-MIBI, ^99m^Tc-tetrofosmin, and ^201^Tl-chloride (Sachdev et al. 1990; Kelly et al. 1993; Maddahi et al. 1994). Unlike SPECT, positron emission tomography (PET) imaging offers several evident advantages of imaging in MPI application including higher spatial resolution, better sensitivity, and an improved attenuation correction. Currently, the used PET radiotracers for MPI studies are [^13^ N]NH_3_, [^15^O]H_2_O, and ^82^Rb (Schelbert et al. 1981; Selwyn et al. 1982; Bergmann et al. 1984). The short half-lives of PET tracers, such as ^15^O (2 min) and ^13^ N (10 min), and the requirement for an on-site cyclotron for manufacturing these tracers are the main restrictions for their usage. Additionally, ^82^Sr/^82^Rb generator is broadly available but it is not an ultimate PET radiotracer because of its high recurring price, very short half-life combined with long positron range that lowers the image resolution. When compared with other PET tracers, fluorine-18 (^18^F) offers suitable nuclear and chemical properties for PET imaging (Okarvi 2001; Varagnolo et al. 2000). Therefore, various ^18^F-labeled radiopharmaceuticals for MPI have been prepared and evaluated and some of these new agents have shown better image quality and a better association to true myocardial blood flow than ^99m^Tc-MIBI (Marshall et al. 2004; Madar et al. 2006; Yu et al. 2007; Huisman et al. 2008; Shoup et al. 2011).

It has been shown that the rhodamine dyes are accumulated in mitochondria and take around 30% of the myocardial intracellular volume in the heart (Kronauge et al. 1992). Thus, numerous ^18^F-rhodamines analogs as potential MPI agents were developed recently (Heinrich et al. 2010; Gottumukkala et al. 2010; Bartholoma et al. 2012). In particular, ^18^F-labeled rhodamine B diethylene glycol ester ([^18^F]RhoBDEGF) has provided an excellent image quality and might be a potential PET tracer for MPI studies (Storey et al. 1993). Recently, our group has developed [^18^F]-FDG-rhodamine, [^124^I]-SIB-rhodamine, and ^68^Ga-NOTA-rhodamine conjugates. These radioconjugates have demonstrated a high myocardial uptake and favorable pharmacokinetics which indicate that some of these radioconjugates may be useful for MPI studies (Aljammaz et al. 2014, 2015a, b, 2019).

The cyclotron-produced positron emitter copper-64 (^64^Cu) together with its 12.7 h half-life and well-known coordination chemistry makes it one of the most attractive radionuclides for PET imaging (McCarthy et al. 1997; Alliot et al. 2011; Szelecsenyi et al. 1993). Therefore, varieties of ^64^Cu-radiolabeled biomolecules for potential use beyond the measurement of glucose metabolism were developed and investigated (Anderson and Ferdani 2009; Zhang et al. 2013; Sprague et al. 2007; Hao et al. 2009; Evangelista et al. 2013). Among these, ^64^Cu-labeled DOTA-somatostatin conjugate (^64^Cu-DOTATATE) has been recently approved by the FDA for the localization of somatostatin receptor-positive neuroendocrine tumors (NETs) in adult patients. For the past several years, we are interested in developing new agents for MPI studies; in this paper, we described the synthesis and initial evaluation of the ^64^Cu-NOTA- and ^64^Cu-NOTAM-rhodamine conjugates.

## Results

### Chemistry

**Scheme 1:**
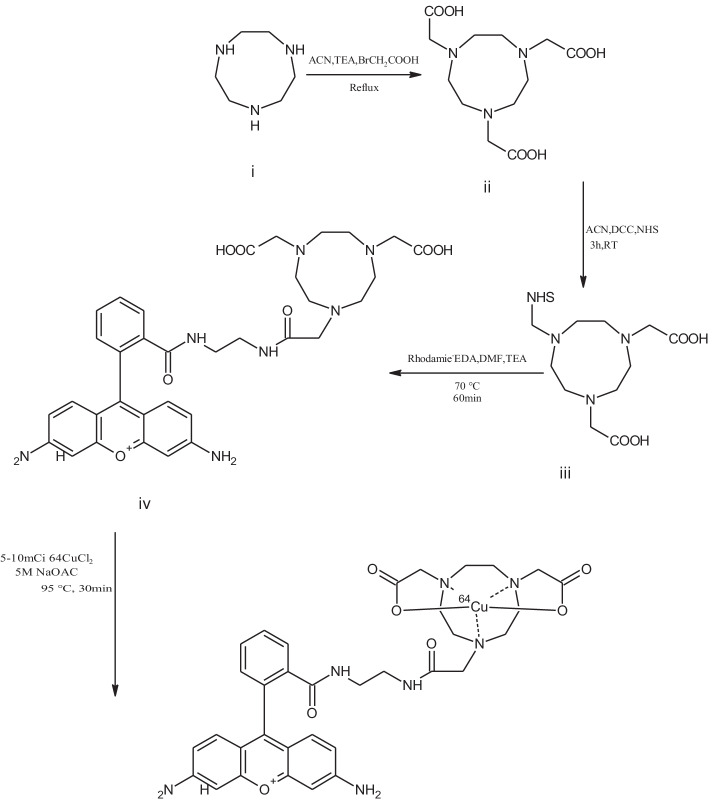
Synthesis of NOTA precursors and ^64^Cu-NOTA-rhodamine conjugate. (i) 1,4,7-Triazacyclononane; (ii) 1,4,7-Triazacyclononane triacetic acid; (iii) *N*-Succinimidyl-1,4,7-triazacyclononane diacetic acid; (iv) NOTA-rhodamine

**Scheme 2:**
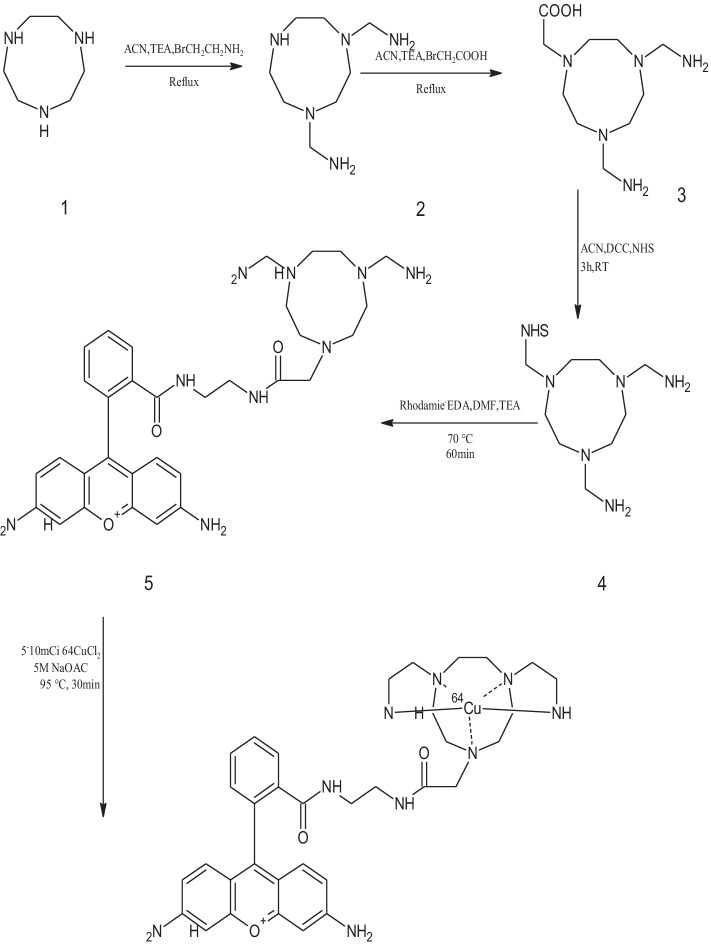
Synthesis of NOTAM precursors and ^64^Cu-NOTAM-rhodamine conjugate. 1,4,7-Triazacyclononane; (2) 1,4,7-Triazacyclononane-*N,N′-*diethylamine (NOTAM); 3) 1,4,7-Triazacyclononane-*N,N′-*diethylamine-*N*′′-acetic acid; (4) *N*-Succinimidyl-1,4,7-triazacyclononane-*N,N′-*diethylamine-*N*′′-acetic acid; (5) NOTAM-rhodamine

The synthetic methods for the preparation of NOTA- and NOTAM-rhodamines are mentioned in Schemes 1 and 2. These conjugates were fully characterized by HPLC and the mass spectral data and agreed with the expected structures. The precursor's NOTA- and NOTAM-rhodamine conjugates were obtained as an off-white precipitate in 60% and 20% yield, respectively. The theoretical calculated molecular masses for NOTA- and NOTAM-rhodamine conjugates were 658 and 628, respectively**.** These values agreed well with the attained ES-MS [M + 1]^+^ = 659 and 629, respectively. Chemical purities for NOTA- and NOTAM-rhodamine conjugates were higher than 98% as assessed by HPLC.

The reference Cu^II^-NOTA- and Cu^II^-NOTAM-rhodamine conjugates were obtained as off-white powders with overall chemical yields of ~ 50%, which appear to decompose beyond 180 °C. The calculated molecular masses for Cu^II^-NOTA- and Cu^II^-NOTAM-rhodamine were 720 and 690, respectively. These values were agreed with the attained ES-MS [M + 1]^+^ = 721 and 691, respectively. Chemical purities of Cu^II^-NOTA- and Cu^II^-NOTAM-rhodamine were higher than 97% as assessed by HPLC with retention times of 11.6 and 13.1 min, respectively.

### Radiochemistry

**Fig. 1:**
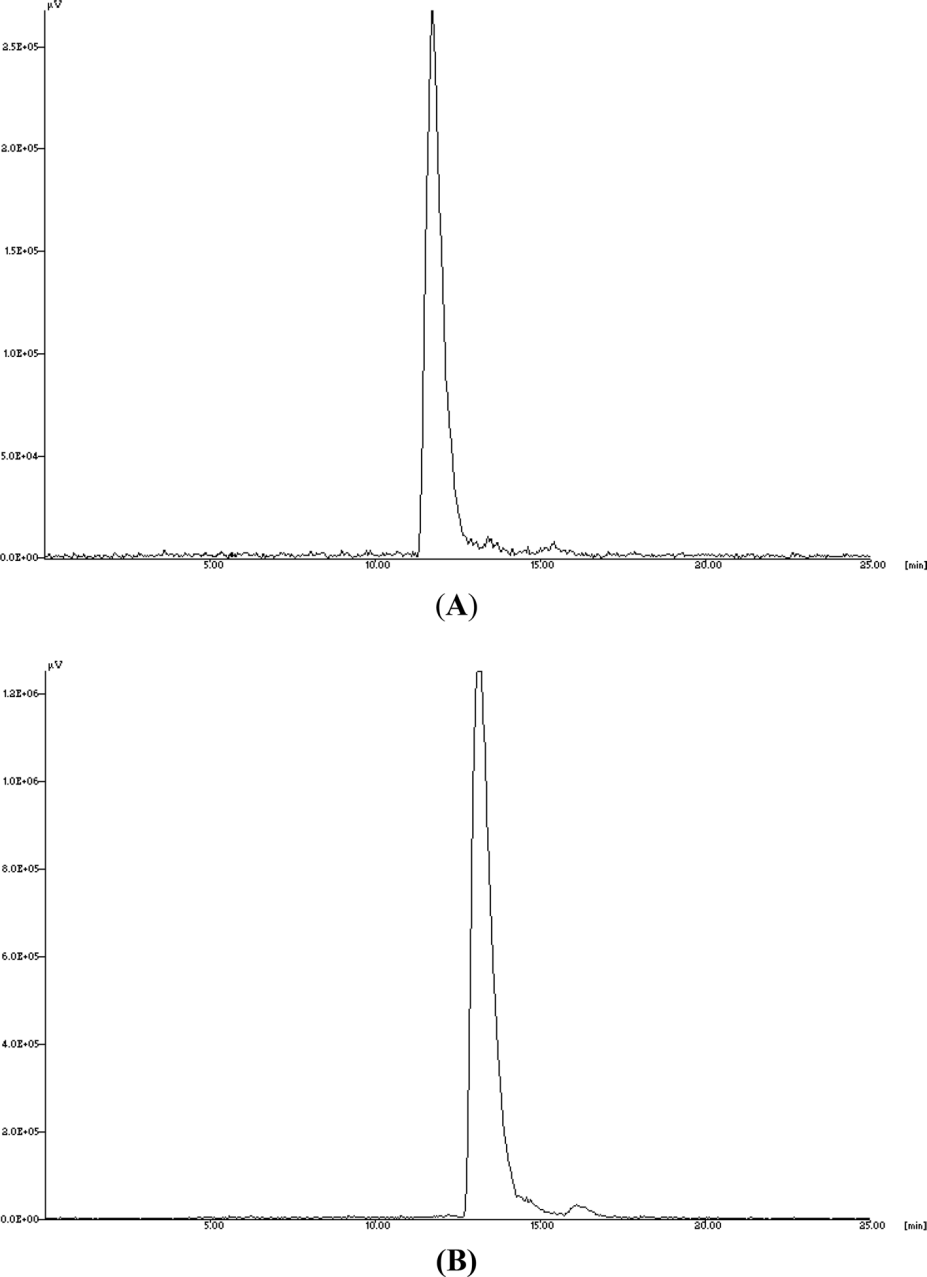
HPLC chromatograms of (**A**) ^64^Cu-NOTA- and (**B**) ^64^Cu-NOTAM rhodamine conjugates

**Fig. 2:**
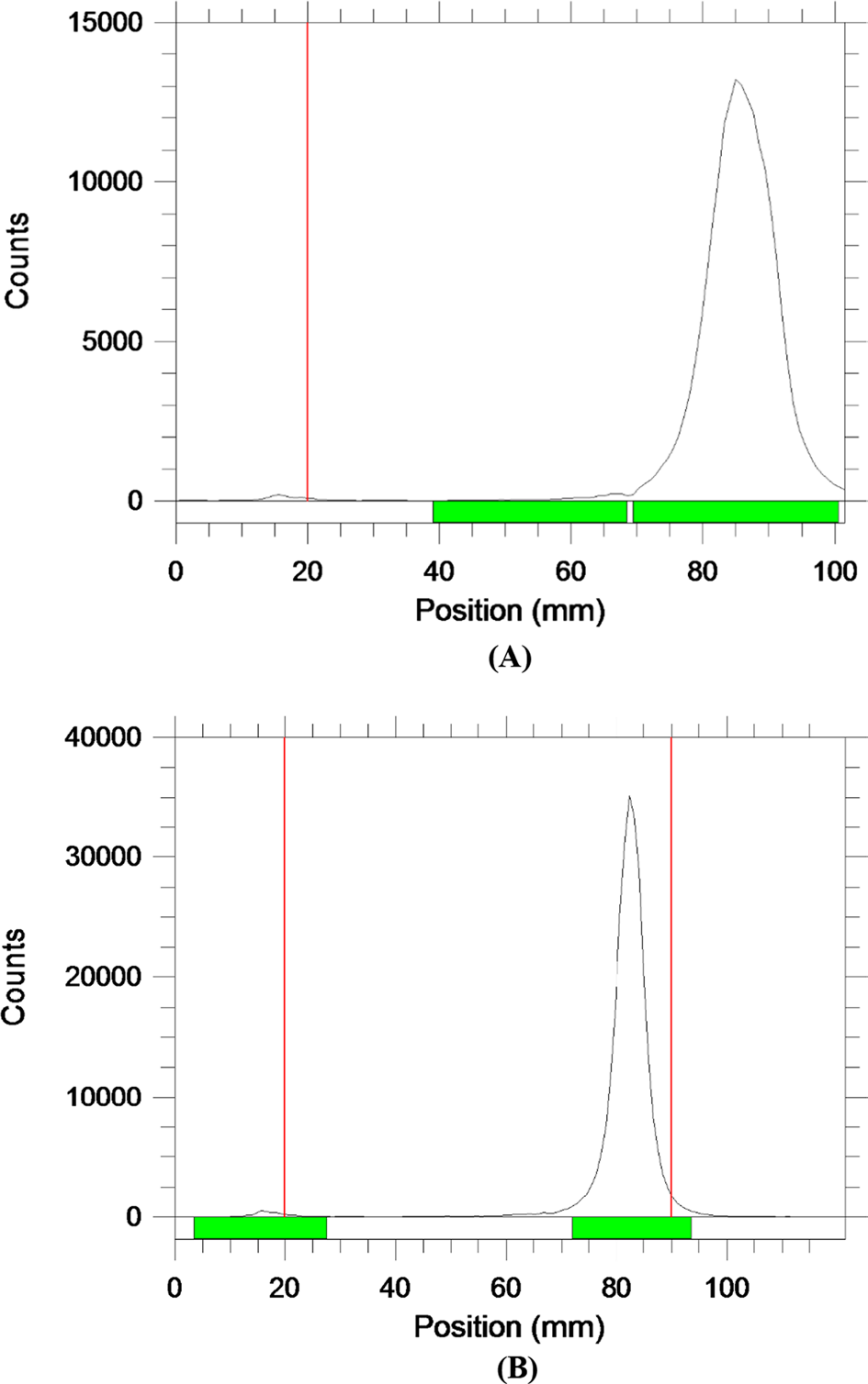
TLC chromatograms of (**A**) ^64^Cu-NOTA- and (**B**) ^64^Cu-NOTAM rhodamine conjugates. In the TLC chromatograms, the free copper remained at the origin (R_*f*_: 0–0.15) while the radiolabeled.^64^Cu-complexes moved to R_f_ values of 0.8–0.95

In an attempt to develop novel PET rhodamine tracers for MPI studies with longer half-life and better pharmacokinetics, we have developed ^64^Cu-NOTA- and ^64^Cu-NOTAM-rhodamine for myocardial PET imaging. The synthetic procedure for the preparation of ^64^Cu-NOTA- and ^64^Cu-NOTAM-rhodamine provided a facile and simple one-step reaction. Radiochemical yields were quantitative (> 95%) in less than 25 min. Radiochemical purities of these radioconjugates were always greater than 98% as determined by HPLC (Fig. 1) and confirmed by TLC. In the TLC chromatograms, the free copper remained at the origin (R_*f*_: 0–0.15) while the radiolabeled complexes had R_f_ values of 0.8–0.95 (Fig. 2).

In addition, the calculated partition coefficient for ^64^Cu-NOTA- and ^64^Cu-NOTAM-rhodamine conjugates were found − 0.57 ± 0.03 and − 0.38 ± 0.04, respectively, representing ~ 41% lower hydrophilic characteristics of the ^64^Cu-NOTA- as compared to the ^64^Cu-NOTAM-rhodamine compound.

### Stability in plasma

The proteolytic degradation of the ^64^Cu-NOTA- and ^64^Cu-NOTAM-rhodamine was calculated in human plasma in vitro. HPLC analysis of the plasma samples revealed that the ^64^Cu-NOTA- and ^64^Cu-NOTAM-rhodamine remained highly stable (> 97%) during incubation at 37 °C for at least 4 h, suggesting a high in vitro stability of these radiolabeled bioconjugates.

### In vivo Biodistribution

Preliminary biological properties of ^64^Cu-NOTA- and ^64^Cu-NOTAM-rhodamine in normal Fischer rats at 60 min p.i. are summarized in Table 1. The results of in vivo biodistribution display rapid and more efficient clearance of ^64^Cu-NOTA-rhodamine from the blood and most of the organs and tissues than ^64^Cu-NOTAM-rhodamine. A high accumulation by the kidneys (0.85 ± 0.41% ID/g) and a low uptake in the liver (0.35 ± 0.06% ID/g) for ^64^Cu-NOTA-rhodamine were observed demonstrating that the main route of elimination was the urinary system. Whereas the moderate uptake of ^64^Cu-NOTAM-rhodamine showed by the liver, spleen, and kidneys (1.19 ± 0.10, 1.19 ± 0.21, and 0.55 ± 0.08% ID/g, respectively) indicates that the route of excretion was predominantly the hepatobiliary and partly urinary systems.

**Table 1:** Biodistribution of ^64^Cu-NOTA- and NOTAM-rhodamine conjugates in normal rats at 60 min post-injection

	^64^Cu-NOTA-rhodamine 1 h	^64^Cu-NOTAM-rhodamine 1 h
Blood	0.42 ± 0.09	0.29 ± 0.05
Lung	0.55 ± 0.12	0.39 ± 0.06
Liver	0.35 ± 0.06	1.19 ± 0.10
Kidney	0.85 ± 0.41	0.55 ± 0.08
Intestine	1.51 ± 0.50	2.64 ± 0.29
Heart	5.60 ± 1.02	9.50 ± 0.99
Muscle	0.90 ± 0.08	1.10 ± 0.09
Spleen	0.91 ± 0.32	1.19 ± 0.21

The values are average of % injected dose/gram ± SD for n = 4

**Fig. 3:**
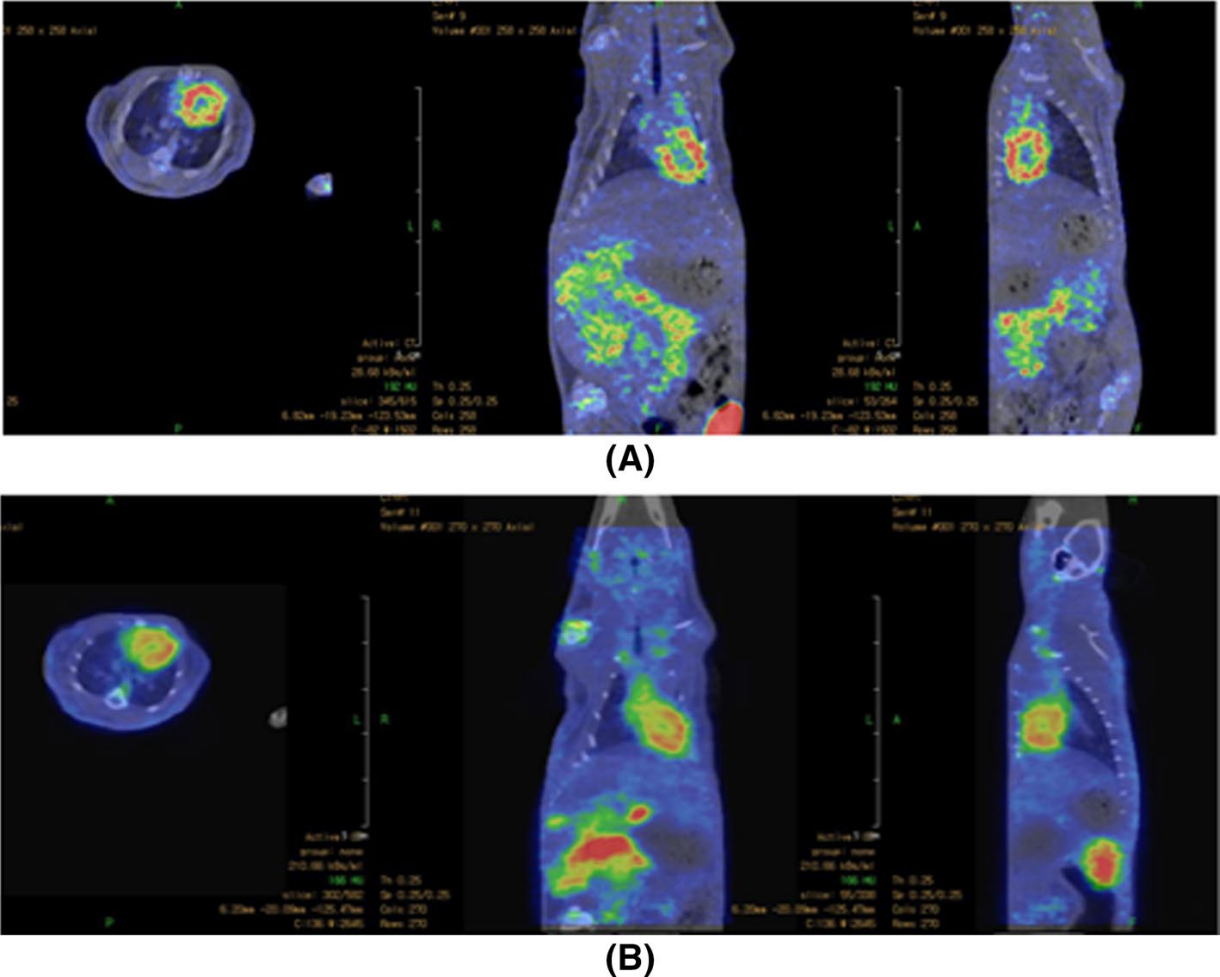
Coronal, transaxial, and sagittal images of normal rats after 60 min post-injection using (**A**) ^64^Cu-NOTA-rhodamine and (**B**) ^64^Cu-NOTAM-rhodamine conjugates (injected dose 7.4 MBq each)

Additionally, the main target organ heart displayed the highest uptake of 9.50 ± 0.99% ID/g of the ^64^Cu-NOTAM-rhodamine, which is higher than the uptake of the ^64^Cu-NOTA-rhodamine conjugate (5.60 ± 1.02% ID/g). Very good heart-to-blood ratios (32.76) and (13.33) were obtained for ^64^Cu-NOTAM- and ^64^Cu-NOTA-rhodamine conjugates, respectively. Initial Nano-PET imaging studies have clearly delineated the heart uptake of ^64^Cu-NOTA- ^64^Cu-NOTAM-rhodamine conjugates with high contrast relative to the background (Fig. 3). These images are concurrent with findings obtained in quantitative biodistribution data reported above.

These results demonstrate that the ^64^Cu-NOTAM-rhodamine conjugate has better heart uptake than the ^64^Cu-NOTA-rhodamine conjugate. However, the latter may pose suitable pharmacokinetic properties over the former and deserve more evaluation. Furthermore, the radioactivity excreted into the urine at the time of sacrifice (60 min p.i.) was collected and checked by radio-HPLC to investigate the in vivo stability of ^64^Cu-NOTA- and ^64^Cu-NOTAM-rhodamine compounds. Radio-HPLC chromatograms of the urine samples displayed that a good amount of radioactivity (> 95%) was still attached to the radiolabeled bioconjugates. These findings show that these radiolabeled bioconjugates are not inclined to fast in vivo degradation and are associated well with the high metabolic stability obtained in human plasma in vitro.

## Discussion

In an attempt to develop novel PET rhodamine tracers for MPI studies with longer half-life and better pharmacokinetics, we have developed ^64^Cu-NOTA- and ^64^Cu-NOTAM-rhodamine for myocardial PET imaging. The Cu^II^ ion is a 3d^9^ with coordination numbers ranging from 4 through 6 it has borderline hardness with high affinity to O and N donor atoms. Therefore, derivatives of TACN with two (NOTA, NOTAM) carboxymethyl pendant arms both complex Cu(II) with good affinity. The former has an N_3_O_3_ donor set that forms a distorted trigonal prismatic geometry. [Cu^II^ NOTA] complex is hexa-coordinated and shows high stability with Log K 21.6. In NOTAM, the presence of more basic donor atoms in the pendant arms has led to an increase in the Log K value of Cu^II^ complexes (Log K 22.4) (Baranyai et al. 2020; Clarke et al. 1990; Clarke and Martell 1991a, b; Wadas et al. 2010; Tolmachev et al. 2017). Therefore, to develop new MPI agents, with suitable characteristics for the PET investigation of myocardial perfusion, we have prepared NOTA- and NOTAM-rhodamine compounds.

^64^Cu-Labeled molecules are promising imaging agents for PET due to the favorable nuclear characteristics of the isotope (t_1/2_ = 12.7 h, *β* + 17.4%, *E*_max_ = 0.656 MeV, *β*^−^ 39%, *E*_max_ = 0.573 MeV) and its availability as no-carrier-added Cu-64. The longer physical half-life of ^64^Cu compared to other PET isotopes enables imaging at delayed time points, which allows sufficient time for clearance from background tissues, resulting in increased image contrast (Banerjee et al. 2014). The synthetic procedure for the preparation of ^64^Cu-NOTA- and ^64^Cu-NOTAM-rhodamine provided a facile and simple one-step reaction, with high radiochemical yield and purity.

The results of in vivo biodistribution display rapid and more efficient clearance of ^64^Cu-NOTA-rhodamine from the blood and most of the organs and tissues than ^64^Cu-NOTAM-rhodamine. A high accumulation by the kidneys and a low uptake in the liver for ^64^Cu-NOTA-rhodamine were observed indicating that the urinary system is the main excretion pathway. While ^64^Cu-NOTAM-rhodamine showed the moderate uptake by the liver, spleen, and kidneys suggesting that the route of excretion was predominantly the hepatobiliary and partly urinary systems. This behavior may be due to the nature of ^64^Cu-NOTA- and ^64^Cu-NOTAM as chelating agents and the overall net charge on ^64^Cu-NOTA- and ^64^Cu-NOTAM-rhodamine molecules (Miao et al. 2006). Additionally, the ^64^Cu-NOTAM-rhodamine displayed the highest uptake in the main target organ heart which is higher than the uptake of the ^64^Cu-NOTA-rhodamine conjugate. These values are similar to heart uptake values obtained from other radiofluorinated rhodamine conjugates reported previously (Gottumukkala et al. 2010; Bartholoma et al. 2012, 2013; Breeman et al. 2005, 2011) and at least two times better than values obtained using ^68^Ga-NOTA- and ^68^Ga-NODAGA-rhodamine conjugates. Additionally, good heart-to-blood ratios of 32.76 for ^64^Cu-NOTAM and 13.33 for ^64^Cu-NOTA-rhodamine conjugates were obtained. The heart-to-blood ratios of the ^64^Cu-NOTAM were found to be superior to the values obtained for other rhodamine conjugates, for example, ^18^F-FDG-rhodamine (28.10) and ^68^Ga-NOTA-rhodamine (4.56) (Aljammaz et al. 2015a, b, 2019), suggesting the usefulness of the ^64^Cu-NOTAM. Moreover, initial Nano-PET imaging studies have clearly delineated the heart uptake of ^64^Cu-NOTA- ^64^Cu-NOTAM-rhodamine conjugates with high contrast relative to the background (Fig. 3). These images are concurrent with finding obtained in quantitative biodistribution data reported above. The data suggest that the ^64^Cu-NOTAM-rhodamine conjugate has better heart uptake properties than the ^64^Cu-NOTA-rhodamine conjugate. But, the latter may pose favorable biokinetics over the former and deserve further investigation. Radio-HPLC chromatograms of the urine samples displayed that a good amount of radioactivity was still attached and these radiolabeled bioconjugates are not inclined to fast in vivo degradation. It is worth mentioning here that when these radiolabeled conjugates were investigated in normal Balb/c mice, nearly no accumulation of ^64^Cu-NOTA- and ^64^Cu-NOTAM-rhodamine in the mice hearts were found, probably due to the in vivo enzymatic breakdown of these radiotracers in mouse serum (data not shown) as observed previously for other rhodamine compounds in mice (Gottumukkala et al. 2010; Aljammaz et al. 2015a, b). These results indicate that mice may not be suitable animal models for the preclinical evaluation of rhodamine conjugates.

## Methods

All chemicals and reagents used in this work were all highest purity grade obtained from commercial sources and were used without further purification unless stated. Acetonitrile (ACN) and dimethylformamide (DMF) were kept over molecular sieves. Sep-Pak cartridges were purchased from Waters-Millipore. Thin-layer chromatography-SG sheets were purchased from Grace Discovery Inc. High-performance liquid chromatography (HPLC) analysis was carried out on Luna; Phenomenex C-18 reversed-phase column (analytical, 250 mm × 4.6 mm). The solvent system used was isocratic (eluant: ACN/H2O, 95/5 with 0.1% TFA at a flow rate of 1.0 mL/min). A Jasco chromatographic system equipped with a variable wavelength ultraviolet monitor and in tandem with a Canberra flow through radioactivity detector was used. Ultraviolet absorption was monitored at 254 nm. Chromatograms were acquired and analyzed using BORWIN software. Elemental analyses were performed on a Perkin Elmer CHN 2400 analyzer. The melting points were measured using a Thomas-Hoover Unimelt capillary melting point apparatus. Mass spectroscopy was performed on Quattra electrospray mass spectrometer.

### Chemistry

#### 1,4,7-Triazacyclononane-*N*,*N*′,*N*″-triacetic acid-rhodamine conjugate (NOTA-rhodamine, iv)

NOTA-rhodamine conjugate was synthesized utilizing the method reported previously (Aljammaz et al. 2014, 2015a, b). In brief, ethylene diamine-rhodamine conjugate (EDA-rhodamine) was dissolved in DMF. To this, triethylamine (TEA, 2 equivalent) and *N*-Succinimidyl-1,4,7-triazacyclononane-*N,N′,N″-*triacetic acid (NOTA-NHS, 1 equivalent) were added. The mixture was shielded from light and stirred at 70 °C for 1 h. The ACN was then added to precipitate the product which was filtered and washed a few times with ACN and dried under vacuum to provide an off-white precipitate compound (Scheme 1). Yield = 57%; Mp = 122–125 °C. C_34_H_40_N_7_O_7_ = MW 658.7

#### 1,4,7-Triazacyclononane-*N*,*N*′-diethylamine (NOTAM, 3)

NOTAM was synthesized by dissolving hydrochloric acid salt of 1,4,7-triazacyclononane (TACN. HCl, 500 mg, 2.1 mmol) in ACN (10 mL) followed by the addition of triethylamine (TEA, 1.16 mL, 8.4 mmol) (Scheme 2). To the stirred mixture, bromoethylamine protected with BOC group (430 mg, 2.1 mmol) in ACN (4 mL) was added and the reaction mixture was then stirred and refluxed for 2 h. The solution was concentrated by rotary evaporation to leave a light yellowish solid product which was washed with ACN (10 mL) to leave a yellowish precipitate. This was then filtered, washed with ACN (10 mL), and dissolved in CH_2_Cl_2/_TFA (1:1, 10 mL) before stirring at room temperature for 30 min for deprotection. The mixture was then dried in vacuo to yield 258 mg (57.1%) of NOTAM as a yellow oily material.C_10_H_25_N_5_ = MW 215.3.

#### 1,4,7-Triazacyclononane-*N*,*N*′-diethylamine-*N*′′-acetic acid (NOTAM-AcOH, 4)

NOTAM (348 mg, 1.42 mmol) was dissolved in ACN (10 mL) followed by the addition of TEA (0.4 mL, 1.42 mmol). To the stirred mixture, bromoacetic acid (BAA, 0.2 g, 1.42 mmol) in ACN (3 mL) was added drop-wise over 5 min. The reaction mixture was then stirred and refluxed for 3 h. The brown solution was concentrated by rotary evaporation to leave a brown solid product which was washed with ACN (8 mL) to leave a creamy precipitate. The off-white precipitate was then filtered, washed with ACN (10 mL), and dried in vacuo to yield 232 mg (59.8%) of NOTAM as an oily material. C_12_H_25_N_2_O_2_ = MW 273.3.

#### *N*-Succinimidyl-1,4,7-triazacyclononane-*N*,*N*′-diethylamine-*N*′′-acetic acid (NOTAM-NHS, 5)

The oily material (120 mg, 0.39 mmol) was dissolved in ACN (6 mL) followed by the addition of NHS (43 mg, 0.39 mmol) and DCC (85 mg, 0.39 mmol). The reaction mixture was stirred at ambient temperature for 3 h. The by-product dicyclohexylurea was then removed by filtration and the filtrate was dried by rotary evaporation to furnish 105 mg (72.7%) of NOTAM-NHS as an oily product. C_16_H_29_N_6_O_4_ = MW 370.

#### 1,4,7-Triazacyclononane-*N*,*N*′-diethylamine-*N*′′-rhodamine (NOTAM-rhodamine conjugate, 6)

For the synthesis of the NOTAM-rhodamine, EDA-rhodamine conjugate (0.15 mmol) in DMF was mixed with TEA (0.30 mmol) and NOTAM-NHS (0.15 mmol). The reaction mixture was allowed to be stirred in dark for 60 min at 70^o^ C. Acetonitrile was added to precipitate the product which was filtered, and washed a few times with ACN. The product was dried under a vacuum to give an off-white precipitate compound. Yield 26%; Mp = 108–110 °C. C_34_H_46_N_9_O_3_ = MW 628.70.

#### Reference Cu^II^-compounds (Cu^II^-NOTA- and Cu^II^-NOTAM-rhodamines)

The Cu-NOTA- and Cu-NOTAM-rhodamine reference compounds were prepared following the procedure reported previously (Aljammaz et al. 2019). In brief, NOTA (5 mg, 7.6 µmol) and NOTAM-rhodamine (5 mg, 7.0 µmol) were allowed to react with an equimolar amount of copper chloride (CuCl_2_) in 0.1% acetic acid in EtOH, 500 µL, pH ~ 4.5) at 95 °C for 30 min. Acetonitrile was added to precipitate the reference compounds which were filtered and washed a few times with ACN. After centrifugation, the compounds were washed a few times and dried under a vacuum to yield products like the yellow powders.

### Radiochemistry

#### ^64^Cu-NOTA- and ^64^Cu-NOTAM-rhodamine compounds

^64^CuCl_2_ was produced by the bombardment of nickel-64 target (^64^Ni, 100 mg ± 10%) for 2 h with 15.5 MeV protons and 100 μA beam current from the Cyclon-30 (IBA) using the ^64^Ni(p,n)^64^Cu nuclear reaction. The irradiated target was dissolved in hydrochloric acid (HCl, 9 M, 8–10 mL) followed by hydrogen peroxide (H_2_O_2_, 0.15 × 1 mL, 30%) with continuous heating (80^o^ C). The dissolved nickel target was transferred in a bottle (150 mL) followed by complete drying then HCl (6 N, 10 mL) was added and passed through an anion exchange column cartridge (TK201, 2 mL) which was preconditioned with HCl (6 M, 5 mL). TK201 cartridge was rinsed with HCl (6 M, 5 mL) to remove traces of ^64^Ni followed by rinsing with HCl (4.5 M, 5 mL) to remove traces of cobalt isotopes. ^64^CuCl_2_ was then eluted with HCl (0.5 M, 10 mL).

The synthetic approach for the preparation of ^64^Cu-NOTA- and -NOTAM-rhodamine conjugates was straightforward. ^64^CuCl_2_ solution (185–370 MBq) was reacted in sealed vials with NOTA- and NOTAM-rhodamine conjugate separately (50 µg each) in sodium acetate buffer (NaOAc, 5 M, pH ~ 4.5, 1.0 mL) at 95 °C for 30 min (Schemes 1, 2). The reaction mixtures were diluted with H_2_O (3 mL), passed through the C18 Sep-Pak cartridge, dried, and finally eluted with ethanol (EtOH, 5 mL). EtOH was then evaporated and the residue was reconstituted with normal saline before passing through a 0.22 μm pore membrane filter for in vitro and in vivo experiments.

### Partition Coefficient

The partition coefficient of ^64^Cu-NOTA- and ^64^Cu-NOTAM-rhodamine conjugates (100 µL, 0.74 MBq each) was determined following the procedure reported previously (Aljammaz et al. 2019). The partition coefficient ^64^Cu-NOTA- and ^64^Cu-NOTAM-rhodamine conjugates was determined by the function: Log_10_ (counts in the octanol layer/counts in the aqueous layer).

### Stability in plasma

^64^Cu-NOTA- and ^64^Cu-NOTAM-rhodamine complexes (0.74 MBq, 100 µL each) were incubated with plasma (200 µL) in duplicate at 37 °C for 2 h. After incubation, proteins were precipitated by ACN/EtOH (400 µL, 1/1 v/v) and centrifuged at 5000 rpm for 5 min. The supernatant phase was filtered and assessed by HPLC following the conditions mentioned above.

### In vivo biodistribution

Animal studies were conducted strictly according to the international regulations and guidelines governing the safe and proper use of laboratory animals. The biodistribution was carried out in normal Fischer male rats (body weight 50–70 g) to determine the in vivo distribution behavior of the ^64^Cu-NOTA- and ^64^Cu-NOTAM-rhodamine conjugates. 100 µL of the radiotracers formulated in saline were injected via the lateral tail vein of rats. Each injected dose contained ~ 2.59 MBq of radioactivity. Rats were sacrificed after 1 h post-injection (p.i.) and tissues/organs of interest were dissected, weighed, and counted for radioactivity. The percent of the injected dose per gram (% ID/g) was measured by counting all tissues in a γ-counter.

### In vivo nano PET/CT imaging

The PET/CT scans were performed using a preclinical NanoPET/CT scanner (Mediso, Hungary) on normal Fischer male rats (body mass 50–75 g). ^64^Cu-NOTA- and ^64^Cu-NOTAM-rhodamine conjugates (100 μL, 7.4 MBq) were injected into each rat through the tail vein and placed in the NanoPET/CT scanner with continuous O_2_ and 2% isoflurane supply. 20 min post tail vein injection of the radiotracers, the rats were imaged for 20 min PET/CT acquisition time. A static scan was acquired at 60 min p.i. The CT scan was performed using the following parameters: X-ray voltage = 50 kVp, Exposure time = 300 ms. A total projection of 288 projects over 360° of rotation was acquired and reconstructed using a cosine filter. This was followed by a PET data acquisition with the following parameters: 5-ns coincidence window and 400–600 keV energy window in 1–5 coincidence mode. Crystal efficiency correction was also applied, with a ring difference of 8, and the images were reconstructed by a three-dimensional ordered-subsets; exception maximum algorithm (subsets, 4; iterations, 6). The pixel size was 0.3 mm. The acquired data in these studies were analyzed by InterVeiw FUSION software developed by Mediso.

### Statistical analysis

Data are expressed as mean ± S.D. where appropriate. For data comparisons, a Student’s *t* test was performed on the mean values using Graph-Pad Software (Graph-Pad Software Inc., San Diego, CA, USA). A probability value of *P* < 0.05 was considered statistically significant.

## Conclusion

In a suitable radiosynthesis approach, ^64^Cu-NOTA- and ^64^Cu-NOTAM-rhodamine compounds were prepared in high radiochemical yields and purities in about 25 min. Preliminary biodistribution in normal Fischer rats at 60 min p.i, exhibited a higher myocardial uptake of ^64^Cu-NOTAM-rhodamine conjugate over the ^64^Cu-NOTA-rhodamine. The data suggest that these radioconjugates may be suitable for MPI studies using PET. However, further evaluation is needed.

## Data Availability

The data associated with this research work are available in this manuscript or upon request from the corresponding author.

## Abbreviations

MPI: Myocardial perfusion imaging
NOTA: 1,4,7-Triazacyclononane-*N,N′,N″-*triacetic acid
NOTAM: 1,4,7-Triazacyclononane-N,N′,diethylamine
PET: Positron emission tomography
SPECT: Single-photon emission computed tomography
^18^F: Fluorine-18
ACN: Acetonitrile
DMF: Dimethylformamide
EtOH: Ethanol
HPLC: High-performance liquid chromatography
TLC: Thin-layer chromatography
